# Expression Pattern and Molecular Mechanism of Oxidative Stress-Related Genes in Myocardial Ischemia–Reperfusion Injury

**DOI:** 10.3390/jcdd10020079

**Published:** 2023-02-13

**Authors:** Jiahe Wu, Jingyi Luo, Huanhuan Cai, Chenze Li, Zhe Lei, Yi Lu, Lihua Ni, Jianlei Cao, Bo Cheng, Xiaorong Hu

**Affiliations:** 1 Department of Cardiology, Zhongnan Hospital of Wuhan University, Wuhan 430071, China; 2 Institute of Myocardial Injury and Repair, Wuhan University, Wuhan 430071, China; 3 Department of Stomatology, Zhongnan Hospital of Wuhan University, Wuhan 430071, China; 4 Department of Nephrology, Zhongnan Hospital of Wuhan University, Wuhan 430071, China

**Keywords:** myocardial ischemia–reperfusion injury, oxidative stress, bioinformatics analysis, enrichment analysis, miRNA, transcription factors, therapeutic drug

## Abstract

(1) Background: The molecular mechanism of oxidative stress-related genes (OSRGs) in myocardial ischemia–reperfusion injury (MIRI) has not been fully elucidated. (2) Methods: Differential expression analysis, enrichment analysis, and PPI analysis were performed on the MIRI-related datasets GSE160516 and GSE61592 to find key pathways and hub genes. OSRGs were obtained from the Molecular Signatures Database (MSigDB). The expression pattern and time changes of them were studied on the basis of their raw expression data. Corresponding online databases were used to predict miRNAs, transcription factors (TFs), and therapeutic drugs targeting common differentially expressed OSRGs. These identified OSRGs were further verified in the external dataset GSE4105 and H9C2 cell hypoxia–reoxygenation (HR) model. (3) Results: A total of 134 DEGs of MIRI were identified which were enriched in the pathways of “immune response”, “inflammatory response”, “neutrophil chemotaxis”, “phagosome”, and “platelet activation”. Six hub genes and 12 common differentially expressed OSRGs were identified. A total of 168 miRNAs, 41 TFs, and 21 therapeutic drugs were predicted targeting these OSRGs. Lastly, the expression trends of Aif1, Apoe, Arg1, Col1a1, Gpx7, and Hmox1 were confirmed in the external dataset and HR model. (4) Conclusions: Aif1, Apoe, Arg1, Col1a1, Gpx7, and Hmox1 may be involved in the oxidative stress mechanism of MIRI, and the intervention of these genes may be a potential therapeutic strategy.

## 1. Introduction

Early successful reperfusion therapy, including thrombolysis or percutaneous coronary intervention (PCI), is a key measure to reduce myocardial infarct size, maintain cardiac ejection function, and improve prognosis in patients with acute myocardial infarction (AMI) [1,2]. However, reperfusion therapy can also cause myocardial ischemia–reperfusion injury (MIRI), resulting in myocardial stunning, apoptosis, necrosis, and arrhythmia [3]. MIRI is associated with a variety of pathophysiological processes, including inflammatory responses, oxidative stress, Ca^2+^ overload, mitochondrial dysfunction, endothelial cell dysfunction, and activation of regulatory cell death modes [4,5]. Studies have shown that MIRI accounts for 50% of the final myocardial infarction size [6,7]. Clinically, there is still a lack of effective prevention and treatment measures for MIRI.

Oxidative stress (OS) is an imbalance between oxidation and antioxidation in the body. The OS process releases a large number of reactive oxygen species (ROS), including superoxide anion, hydroxyl radical, and hydrogen peroxide [8]. Excessive ROS induces oxidative damage to proteins, lipids, and DNA, generates a large number of oxidative intermediates, increases neutrophil infiltration and protease secretion, activates the regulatory cell death process, and ultimately leads to tissue damage [9]. Evidence suggests that the OS process is involved in coronary atherosclerosis [10], diabetic cardiomyopathy [11], hypertrophic cardiomyopathy [12], AMI [13], and many other cardiovascular diseases.

The mechanism of OS involved in MIRI has been extensively studied. Studies have shown that the activation of ERK1/2, JAK2/STAT3 pathway, and Akt/eNOS pathway can inhibit OS and reduce MIRI [14,15]. NF-κB promotes OS-induced necrosis and MIRI by inhibiting the Nrf2 ARE pathway [16]. PHLDA3 protects against MIRI by inhibiting OS and inflammatory responses via the Akt/Nrf2 axis [17]. LRP6 protects the heart from OS through the interaction of HSF1 and GSK3β [18]. Dexmedetomidine reverses HR-induced OS in cardiomyocytes through the SIRT1/CHOP signaling pathway [19]. Dapagliflozin alleviates hypoxia/reoxygenation-induced cardiac dysfunction and oxidative damage by regulating AMPK [20]. These studies have shown a close correlation between OS and the progression of MIRI, and the injury can be effectively inhibited by inhibiting the OS process. Despite these findings, the expression pattern and mechanism of oxidative stress-related genes (OSRGs) in MIRI have not been fully elucidated.

In this study, we used bioinformatics methods to perform data mining on the cardiac tissue transcriptome sequencing datasets of the myocardial ischemia–reperfusion model. Datasets GSE160516, GSE61592, and GSE4105 were downloaded and then analyzed to screen differentially expressed OSRGs and study their mechanisms. miRNAs, TFs, and therapeutic drugs targeting these OSRGs were predicted using relevant databases. This study provides new insights into the molecular mechanisms of OS involved in MIRI, aiming to understand the development of this biological process and discover new therapeutic drugs.

## 2. Materials and Methods

### 2.1. Data Resource

The MIRI-related mRNA microarray datasets GSE160516 (mouse, mRNA), GSE61592 (mouse, mRNA), and GSE4105 (rat, mRNA) were obtained from Gene Expression Omnibus (GEO) database (https://www.ncbi.nlm.nih.gov/, accessed on 2 August 2022). GSE160516 comes from the GPL23038 platform [Clariom_S_Mouse] Affymetrix Clariom S Assay, Mouse (Includes Pico Assay). GSE61592 comes from the GPL6887 platform Illumina MouseWG-6 v2.0 expression beadchip. GSE4105 comes from the GPL341 platform [RAE230A] Affymetrix Rat Expression 230A Array. Animals in all three datasets were subjected to myocardial ischemia–reperfusion (IR) treatment. In GSE160516, wildtype male mice (10 weeks old) were subjected to 30 min of ligation of the left anterior descending coronary artery (LAD) followed by the reperfusion of 6, 24, and 72 h. Mice in the control group were subjected to sham surgery. Each group contained four mice. In GSE61592, there were three mice in each group. In the experimental group, the LAD was occluded for 90 min followed by 72 h of reperfusion. In GSE4105, each group contained three sham operation mice and three myocardial ischemia–reperfusion model rats. In the experimental group, the LAD was occluded for 30 min followed by 2 days and 7 days of reperfusion. GSE160516 and GSE61592 were used as the analysis datasets, and GSE4105 was used as the external validation dataset for further bioinformatics analysis.

### 2.2. Identification of the DEGs in Myocardial Ischemia-Reperfusion Injury

Raw expression data of datasets GSE160516 and GSE61592 were downloaded from the GEO database, and the “limma package” of R software (version 4.0.1) was used to screen differentially expressed genes (DEGs) between reperfusion (myocardial tissue with a reperfusion time of 72 h) and sham myocardium [21]. The *p*-value was corrected by the Benjamini–Hochberg method. The screening threshold of DEGs was an adjusted *p*-value <0.05 and |logFC| >2. Genes differentially expressed in both datasets were considered to be common DEGs.

### 2.3. Identification of DEGs Associated with Oxidative Stress

The Molecular Signatures Database (MSigDB, http://www.gsea-msigdb.org/gsea/msigdb/index.jsp, accessed on 10 August 2022) was used to retrieve the OS-related items and download the OSRGs. Finally, 424 OSRGs were selected as candidate genes in the subsequent analysis (Supplementary file S1). Differentially expressed OSRGs were obtained by the intersection of DEGs and candidate OSRGs using Online Venn diagram website (http://bioinformatics.psb.ugent.be/webtools/Venn/, accessed on 11 August 2022).

### 2.4. GO Annotation and KEGG Pathway Enrichment Analysis

The “cluster Profiler package” of R software (version 4.0.1) was used to perform Gene Ontology (GO) annotation and Kyoto Encyclopedia of Genes and Genomes (KEGG) pathway enrichment analysis for common DEGs and differentially expressed OSRGs [22]. The GO annotation includes biological process (BP) analysis, cellular component (CC) analysis, and molecular function (MF) analysis. The enrichment analysis results were screened with *p* < 0.05 as the threshold.

### 2.5. Construction of the Protein–Protein Interaction Network and Identification of Hub Genes

The STRING (https://cn.string-db.org, accessed on 30 August 2022) online database was used to evaluate and integrate the interactions of proteins expressed by common DEGs or differentially expressed OSRGs. Cytoscape software (version 3.9.0) was used to visualize the analysis results and construct the corresponding protein-protein interaction (PPI) networks. Through the Cytohubba plugin of Cytoscape software, four algorithms (MCC, Degree, EPC, and Betweenness) were used for further analysis of the PPI network, and the top 10 genes were screened. Genes predicted by three or more algorithms at the same time were considered hub genes.

### 2.6. Validation of the Relationship between Common Differentially Expressed OSRGs and Reperfusion Time

GSE160516 contains gene expression data from reperfusion myocardial tissue at 6, 24, and 72 h after 30 min of ischemia in mice. on the basis of the original gene expression data in GSE160516, the relationship between common differentially expressed OSRGs expression and reperfusion time was further studied.

### 2.7. Prediction of OSRG-Related miRNAs, Transcription Factors, and Targeted Therapeutic Drugs

Online databases miRDB (https://mirdb.org/, accessed on 31 August 2022), Targetscan (https://www.targetscan.org/vert_72/, accessed on 2 September 2022), miRWalk (http://mirwalk.umm.uni-heidelberg.de/, accessed on 2 September 2022), microT-CDS (https://dianalab.e-ce.uth.gr/html/dianauniverse/index.php?r=microT_CDS, accessed on 2 September 2022), TarBase v.8 (https://dianalab.e-ce.uth.gr/html/diana/web/index.php?r=tarbasev8, accessed on 2 September 2022), and miRNet (https://www.mirnet.ca/, accessed on 3 September 2022) were used to predict miRNAs targeting common differentially expressed OSRGs [23,24]. miRNAs predicted by three or more databases at the same time were considered to target this gene. The online databases miRNet and TRRUST (https://www.grnpedia.org/trrust/, accessed on 2 October 2022) were performed to predict common TFs of differentially expressed OSRGs [25]. TFs predicted by both databases were considered to be the TFs of the gene. The DGIDB database (https://dgidb.org/, accessed on 2 October 2022) and CTD database (http://ctdbase.org/, accessed on 2 October 2022) were used to predict therapeutic drugs targeting common differentially expressed OSRGs, and the drugs that predicted by both databases were considered to be therapeutic drugs of the gene [26]. Drugs that simultaneously targeted more than two genes were considered potential therapeutic agents for MIRI. According to the molecular regulatory relationships predicted above, Cytoscape software was used to visualize the predicted results and draw the network diagram.

### 2.8. Further Validation of Common Differentially Expressed OSRGs in External Dataset GSE4105

GSE4105 contains gene expression data from reperfusion myocardial tissue in rats at 2 days and 7 days after 30 min of ischemia. On the basis of the original expression data of genes in the external validation dataset GSE4105, Graphpad Prism software (version: 9.0) was used to further verify the expression of common differentially expressed OSRGs. An independent-sample *t*-test was used to compare the two groups to analyze the difference in gene expression between reperfusion and sham myocardium, and *p* < 0.05 was considered statistically significant.

### 2.9. Establishment of Hypoxia–Reoxygenation Model in H9C2 Myocardial Cell Line

The rat ventricular myocyte line H9C2 was purchased from BeNa Culture Collection (Beijing, China). Cells were cultured at 37 °C in a 5% CO_2_ incubator in Dulbecco’s modified Eagle’s medium (DMEM, Gibco, Invitrogen, Carlsbad, CA, USA) containing 10% fetal bovine serum (FBS, Gibco, Australia) and 1% penicillium–streptomycin (Sigma-Aldrich, St. Louis, MO, USA). The three-gas incubator was used to construct a hypoxic environment (1% O_2_, 5% CO_2_, and 94% N_2_). When H9C2 cells grew to an appropriate density, they were cultured under hypoxia for 24 h, and then replaced with a new medium and cultured under normoxia for 6 h to establish the cardiomyocyte hypoxia–reoxygenation (HR) model. The medium for H9C2 cells in the control group was changed at the same time as that in the HR group, and the cells were always cultured under normoxic conditions (21% O_2_, 5% CO_2_, and 74% N_2_).

### 2.10. Real-Time Quantitative Polymerase Chain Reaction (RT-qPCR)

Cell samples were sequentially subjected to total RNA extraction and reverse transcription using FastPure^®^ Cell/Tissue Total RNA Isolation Kit V2 (Vazyme, Nanjing, China) and Hifair^®^ Ⅲ 1st Strand cDNA Synthesis SuperMix for qPCR (Ye Sen, Shanghai, China) according to the manufacturer’s protocol. The Bio-Rad Connect Real-time PCR Detection System was performed to carry out the qRT-PCR, using Hieff UNICON^®^ Universal Blue qPCR SYBR Green Master Mix (Ye Sen, Shanghai, China). β-Actin was used as the internal reference to compare the expression differences of common differentially expressed OSRGs between the control and HR groups. The changes were calculated using the 2^−∆∆Ct^ method. Supplementary File S2 shows the details of the corresponding primers. Data were presented as the mean values ± standard error of the mean (SEM) from at least three independent experiments. An independent-sample *t*-test was used to compare gene expression differences between the control and HR groups. A *p*-value <0.05 was considered statistically significant.

## 3. Results

### 3.1. Identification of DEGs and Differentially Expressed OSRGs in Myocardial Ischemia-Reperfusion Injury

**Figure 1:**
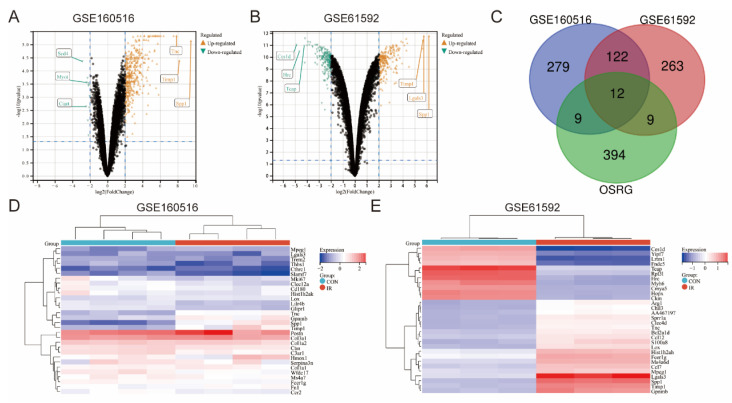
Identification of DEGs and differentially expressed OSRGs in myocardial ischemia–reperfusion injury. (**A**) The volcano plot of GSE160516. (**B**) The volcano plot of GSE61592. For (A,B), the blue dots in the upper-left box are downregulated genes, the orange-yellow dots in the upper-right box are upregulated genes, and the black dots represent genes that are not significantly different. (**C**) Venn diagram of DEGs in GSE160516, GSE61592, and OSRGs in MSigDB. (**D**) Heatmap of the top 30 DEGs in GSE160516. (**E**) Heatmap of the top 30 DEGs in GSE61592.

Differential expression analysis was performed on MIRI-related datasets GSE160516 and GSE61592 to screen DEGs between MIRI and sham myocardial tissues. On the basis of the screening threshold mentioned above, 422 DEGs were identified in GSE160516, of which 411 were upregulated and 11 were downregulated; 406 DEGs were identified in GSE61592, of which 219 were upregulated and 187 were downregulated. Supplementary File S3 presents the detailed results of the differential expression analysis. Figure 1A,B are volcano plots showing the results of differential expression analysis in GSE160516 and GSE61592. The DEGs in these two datasets and the candidate OSRGs in the MSigDB database were intersected to obtain the common DEGs and differentially expressed OSRGs (Figure 1C). A total of 134 common DEGs were identified in GSE160516 and GSE61592, and 21 OSRGs were screened out in each dataset. Twelve OSRGs (Aif1 (allograft inflammatory factor 1), Apoe (apolipoprotein E), Arg1 (arginase 1), Cdk1 (cyclin-dependent kinase 1), Col1a1 (collagen, type I, alpha 1), Gpx1 (glutathione peroxidase 1), Gpx7 (glutathione peroxidase 7), Hmox1 (heme oxygenase 1), Hp (haptoglobin), Mmp14 (matrix metallopeptidase 14), Sirpa (signal-regulatory protein alpha), and Trem2 (triggering receptor expressed on myeloid cells 2)) were significantly differentially expressed in both datasets. Figure 1D presents the heatmap of the top 30 genes with differential expression fold in GSE160516. Figure 1E shows the heatmap of the top 30 DEGs in GSE61592.

### 3.2. Enrichment Analysis of Common DEGs in GSE160516 and GSE61592

**Figure 2:**
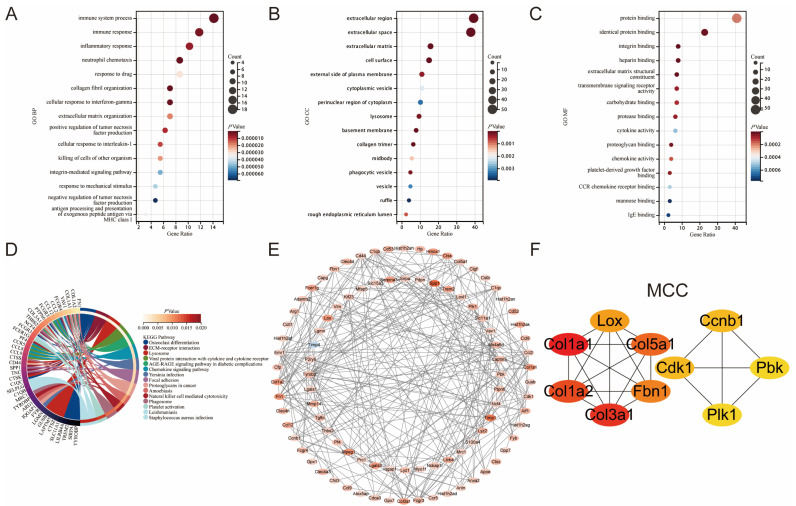
Enrichment analysis and PPI analysis of CoDEGs in GSE160516 and GSE61592. (**A**) Bubble plot of the top 15 biological processes. (**B**) Bubble plot of the top 15 cellular components. (**C**) Bubble plot of the top 15 molecular functions. (**D**) Circle plot of the top 15 KEGG pathway enrichment analysis. (**E**) The whole PPI Network of CoDEGs. The shade of the color represents the magnitude of the relative change multiple in GSE160516. (**F**) Prediction results of the MCC algorithm. A darker color denotes a higher score on this algorithm.

GO annotation and KEGG pathway enrichment analysis were performed for common DEGs in datasets GSE160516 and GSE61592. Figure 2A–D presents the top 15 ranked terms or pathways in the enrichment analysis results. BP analysis of GO annotation showed that common DEGs in MIRI were mainly enriched in the terms of “immune system process”, “inflammatory response”, “neutrophil chemotaxis”, “collagen fibril organization”, “positive regulation of tumor necrosis factor production”, etc. (Figure 2A). CC analysis of GO annotation showed that common DEGs were mainly located in the “extracellular region”, “cell surface”, “cytoplasmic vesicle”, “lysosome”, “phagocytic vesicle”, etc. (Figure 2B). For GO MF analysis, common DEGs were mainly enriched in the terms of “protein binding”, “integrin binding”, “transmembrane signaling receptor activity”, “cytokine activity”, “chemokine activity”, etc. (Figure 2C). In addition, “ECM–receptor interaction”, “lysosome”, “chemokine signaling pathway”, “natural killer cell-mediated cytotoxicity”, “phagosome”, and “platelet activation” were the main pathways in the KEGG enrichment analysis results (Figure 2D).

### 3.3. Construction of the Protein-Protein Interaction Network and Identification of Hub Genes in Myocardial Ischemia-Reperfusion Injury

Protein interaction analysis of common DEGs was performed using the STRING database, and the results were visualized using Cytoscape software. A PPI network with 94 nodes and 252 edges was constructed (Figure 2E). The depth of color in this network plot represents the magnitude of the differential expression fold in GSE160516. Four algorithms (MCC, Degree, EPC, and Betweenness) were used to screen the hub genes in this network. Finally, Aif1, Cd44 (CD44 antigen), Col1a1, Emr1 (Emerin homolog 1), Fn1 (fibronectin 1), and Tyrobp (TYRO protein tyrosine kinase binding protein) were identified as the hub genes of MIRI. Figure 2F shows the screening result of the MCC algorithm. The screening results of the other three algorithms are presented in Supplementary File S4.

### 3.4. Expression Pattern of OSRGs in GSE160516 and GSE61592

**Figure 3:**
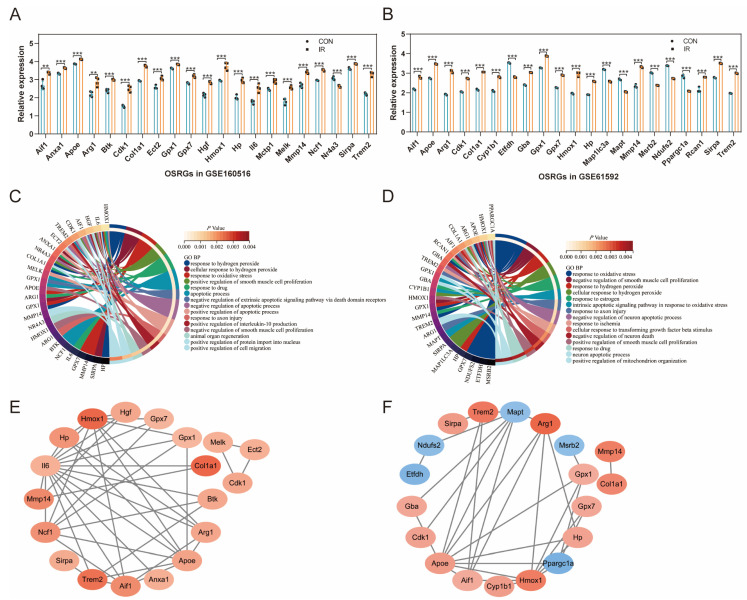
Expression pattern of OSRGs in cardiac ischemia-reperfusion injury. (**A**) Expression pattern of OSRGs in GSE160516. (**B**) Expression pattern of OSRGs in GSE61592. (**C**) Circle plot of Go BP analysis for OSRGs in GSE160516. (**D**) Circle plot of Go BP analysis for OSRGs in GSE61592. (**E**) PPI network of OSRGs in GSE160516. (**F**) PPI network of OSRGs in GSE61592. For panels (**A**,**B**), ** *p* < 0.01, *** *p* < 0.001.

The expression pattern of differentially expressed OSRGs was analyzed on the basis of the original gene expression data in GSE160516 and GSE61592. As shown in Figure 3A, 21 OSRGs were significantly differentially expressed in GSE160516, of which 20 were upregulated and one was downregulated. Figure 3B shows 21 significantly differentially expressed OSRGs in GSE61592, of which 15 were upregulated and six were downregulated. BP analysis of GO annotation showed that the OSRGs in GSE160516 were mainly enriched in the terms of “response to hydrogen peroxide”, “response to oxidative stress”, “positive regulation of smooth muscle cell proliferation”, “apoptotic process”, “positive regulation of cell migration”, etc. (Figure 3C); OSRGs in GSE61592 were mainly enriched in the terms of “response to oxidative stress”, “negative regulation of smooth muscle cell proliferation”, “response to hydrogen peroxide”, “response to estrogen”, “intrinsic apoptotic signaling pathway in response to oxidative stress”, etc. (Figure 3D). Figure 3E is the PPI network of OSRGs in GSE160516 (19 nodes and 42 edges). Figure 3F is the PPI network of OSRGs in GSE61592 (19 nodes and 31 edges). The color depth of these two networks represents the magnitude of the respective differential expression fold in the two datasets.

### 3.5. Validation of the Relationship between Common Differentially Expressed OSRGs and Reperfusion Time

**Figure 4:**
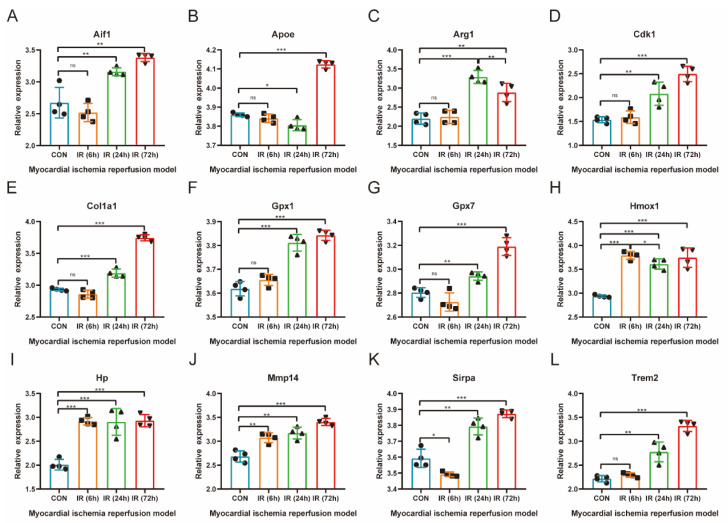
Validation of the relationship between common differentially expressed OSRGs and reperfusion time. The mice were divided into the control group and 6, 24, and 72 h reperfusion groups (*n* = 4 each group). For panels (**A**–**L**), ns indicates no statistical difference; * *p* < 0.05, ** *p* < 0.01, *** *p* < 0.001.

The gene expression data in GSE160516 were evaluated to compare the relationship of common differentially expressed OSRGs with reperfusion time (Figure 4A–L). These genes generally showed an increasing trend in the myocardial ischemia–reperfusion model. The expression of Apoe decreased within 24 h of reperfusion and then increased. The expression of Arg1 increased within 24 h of reperfusion and then decreased. Hmox1, Hp, Mmp14, and Sirpa showed differential expression at 6 h after reperfusion, in which the expressions of Hmox1, Hp, and Mmp14 were increased, while the expression of Sirpa decreased first and then increased.

### 3.6. Prediction of OSRGs-Related miRNAs, Transcription Factors, and Therapeutic Drugs

**Figure 5:**
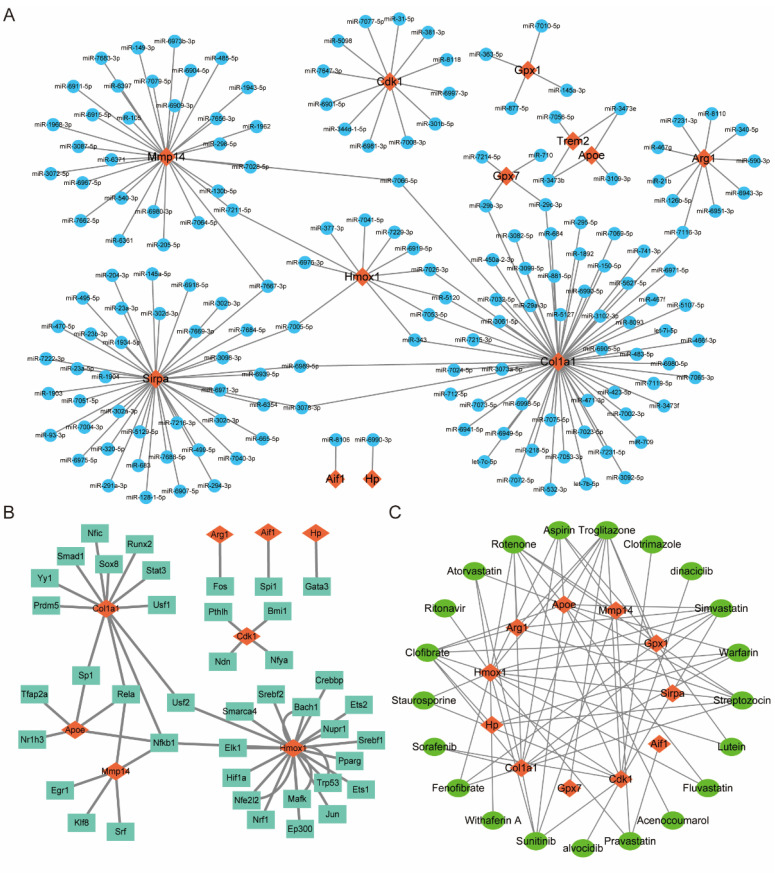
Prediction of OSRG-related miRNAs, transcription factors, and therapeutic drugs. (**A**) The miRNA–mRNA regulatory network. (**B**) The TF–mRNA regulatory network. (**C**) The drug–mRNA regulatory network. For panels (**A**–**C**), orange-red diamond patterns represent OSRGs; blue circular patterns represent miRNAs; cyan rectangular patterns represent transcription factors; green oval patterns represent the therapeutic drug.

Relevant databases were used to predict miRNAs, transcription factors, and therapeutic drugs of common differentially expressed OSRGs. Cytoscape software was used to visualize the predicted results and draw the network diagrams (Figure 5A–C). A total of 168 miRNAs were predicted to target these OSRGs, and an miRNA–mRNA regulatory network with 180 nodes and 183 edges was constructed (Figure 5A). Among the predicted miRNAs, miR-29b-3p, miR-29c-3p, miR-3078-3p, miR-343, miR-3473b, miR-3473e, miR-5120, miR-6989-5p, miR-7005-5p, miR-7026-3p, miR-7053-5p, miR-7066-5p, miR-7116-3p, miR-7211-5p, and miR-7667-3p target two or more OSRGs simultaneously. A total of 41 TFs were predicted to target these OSRGs, and a TF-mRNA regulatory network with 49 nodes and 52 edges was constructed (Figure 5B). Among the predicted TFs, Nfkb1 (nuclear factor kappa B subunit 1), Rela (RELA proto-oncogene), Sp1 (Sp1 transcription factor), and Usf2 (upstream transcription factor 2) target two or more OSRGs simultaneously. In addition, 21 drugs, such as ritonavir, staurosporine, simvastatin, lutein, aspirin, warfarin, and clofibrate, may be therapeutic agents for MIRI. Figure 5C is the corresponding drug–mRNA regulatory network (32 nodes and 71 edges).

### 3.7. Further Validation of Common Differentially Expressed OSRGs in External Dataset GSE4105

**Figure 6:**
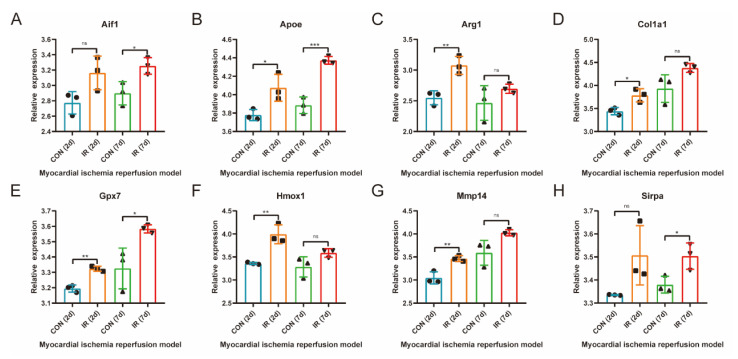
Further validation of common differentially expressed OSRGs in external dataset GSE4105. Gene expression changes were observed at 2 and 7 days of reperfusion in rats (*n* = 3 each group). For panels (**A**–**H**), ns indicates no statistical difference; * *p* < 0.05, ** *p* < 0.01, *** *p* < 0.001.

The expression of common differentially expressed OSRGs was further verified in the external dataset GSE4105. As demonstrated in Figure 6A–H, eight genes (Aif1, Apoe, Arg1, Col1a1, Gpx7, Hmox1, Mmp14, and Sirpa) were significantly differentially expressed in the rat myocardial ischemia-reperfusion model. Apoe, Arg1, Col1a1, Gpx7, Hmox1, and Mmp14 significantly increased on the second day of reperfusion, and Aif1, Apoe, Gpx7, and Sirpa significantly increased on the seventh day of reperfusion.

### 3.8. qRT-PCR Verification of OSRGs in H9C2 Cell Hypoxia–Reoxygenation Model

**Figure 7:**
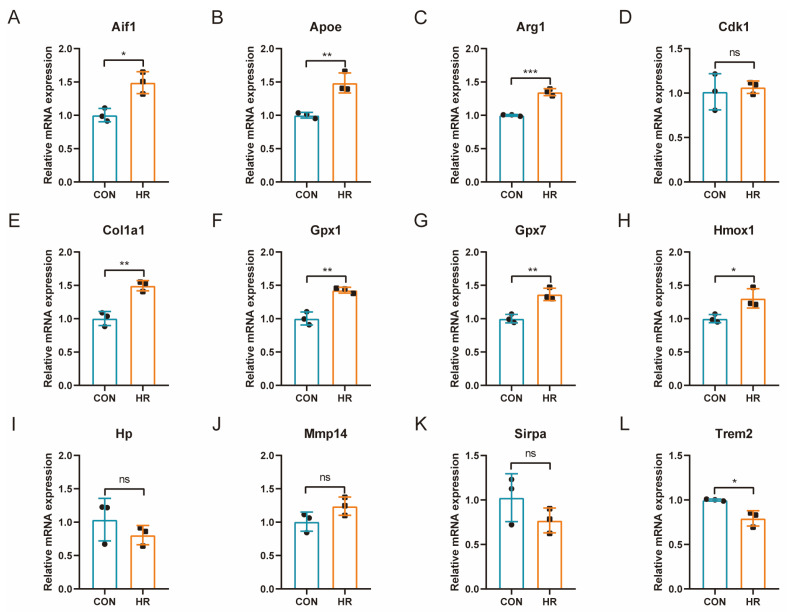
qRT-PCR Verification of common differentially expressed OSRGs in H9C2 cell hypoxia–reoxygenation model. For panels (**A**–**L**), ns denotes no statistical difference; * *p* < 0.05, ** *p* < 0.01, *** *p* < 0.001.

The gene expression of common differentially expressed OSRGs was further verified in the H9C2 cardiomyocytes HR model (Figure 7A–L). The results showed that a total of eight genes were significantly differentially expressed, of which Aif1, Apoe, Arg1, Col1a1, Gpx1, Gpx7, and Hmox1 were significantly upregulated and Trem2 was significantly downregulated. The changing trend of seven highly expressed genes was consistent with the prediction results, whereas Trem2 showed an opposite trend. Enrichment analysis of the differentially expressed genes in the qPCR results showed that these genes were mainly involved in the “response to oxidative stress” (Gpx1, Gpx7, Hmox1, and Apoe), “negative regulation of smooth muscle cell proliferation” (Hmox1, Apoe, and Aif1), “response to hydrogen peroxide” (Col1a1, Gpx1, and Hmox1), and “lipoprotein particle binding” (Trem2 and Apoe) pathways.

## 4. Discussion

Reperfusion therapy after myocardial ischemia is the most effective way to rescue patients with AMI; however, reperfusion therapy can also cause damage to myocardial cells [26]. OS is one of the important pathogeneses of MIRI, but the expression pattern and molecular regulation mechanism of OSRGs in this biological process have not been elucidated [18].

In this study, bioinformatics methods were used to perform data mining on MIRI-related transcriptome microarray datasets, extract relevant biological information from high-dimensional data, and find the key pathways and OSRGs that affect the occurrence of MIRI. This approach is a new trend in disease research in the era of information medicine. Finally, six hub genes and 12 common differentially expressed OSRGs were identified in the analyzed datasets. A total of 168 miRNAs, 41 TFs, and 21 therapeutic drugs were predicted to target these OSRGs. The expression trends of Aif1, Apoe, Arg1, Col1a1, Gpx7, and Hmox1 were confirmed in the external dataset and HR model. These identified molecules have the potential to be used as biomarkers of MIRI for the specific treatment of patients and to improve MIRI in diagnosis, treatment, and prevention.

Through the bioinformatics approach, 134 common DEGs were screened out between MIRI model tissue and sham-operated myocardial tissue in mice. Enrichment analysis showed that these genes were involved in the “immune response”, “inflammatory response”, “neutrophil chemotaxis”, “lysosome”, “chemokine signaling pathway”, “phagosome”, “platelet activation”, and other biological processes or pathways. Inflammation is a key part of the MIRI process. These pathways have been confirmed by related studies. During MIRI, TLRs recognize damage-associated molecular patterns (DAMPs), activate downstream signaling pathways such as the NF-κB signaling pathway, and induce inflammatory responses [27,28]. MIRI aggravates the expression of CXC chemokines, as well as induces monocyte infiltration and macrophage activation [29]. Autophagy is a biological process via which cells degrade themselves to maintain homeostasis of life activities, which is closely related to MIRI. During reperfusion, LC3-II and SQSTM1 protein levels increase, the process of fusion between autophagosomes and lysosomes decreases, and autophagic flux is blocked [30]. Platelet-derived serotonin induces neutrophil degranulation to release myeloperoxidase and hydrogen peroxide (H_2_O_2_), resulting in increased infarct inflammation and reduced myocardial salvage [31].

Further PPI analysis identified six hub genes (Aif1, Cd44, Col1a1, Emr1, Fn1, and Tyrobp) in MIRI. Aif1 may be released after cardiac IR, leading to Toll-like receptor (TLR)-mediated activation of macrophages and dendritic cells, thereby resulting in cytokine production and activation of immune responses [32]. Cd44 was localized to infiltrating leukocytes, wound myofibroblasts, and vascular cells. Compared with wildtype mice, Cd44 (−/−) animals showed enhanced neutrophil and macrophage infiltration and increased expression of proinflammatory cytokines in MIRI [33,34]. Col1a1 and Fn1 are markers of the extent of fibrosis and represent the extent of repair of damaged myocardial tissue. Tyrobp is expressed in circulating immune cells and is a cell membrane-associated protein. It has been reported to be involved in ischemia–reperfusion injury in liver, lung, and kidney [35]. These results suggest a link between the predicted hub genes and MIRI, and further investigation of the mechanism is needed.

Twelve common differentially expressed OSRGs (Aif1, Apoe, Arg1, Cdk1, Col1a1, Gpx1, Gpx7, Hmox1, Hp, Mmp14, Sirpa, and Trem2) were identified in GSE160516 and GSE61592. A total of 168 miRNAs and 41 TFs targeting these OSRGs were predicted by related databases. miRNAs miR-29b-3p, miR-29c-3p, miR-3078-3p, miR-343, miR-3473b, miR-3473e, miR-5120, miR-6989-5p, miR-7005-5p, miR-7026-3p, miR-7053-5p, miR-7066-5p, miR-7116-3p, miR-7211-5p, and miR-7667-3p target two or more OSRGs simultaneously. Nfkb1, Rela, Sp1, and Usf2 target more than two OSRGs simultaneously and are considered as TFs for OS processes. Previous studies have shown that miR-29b-3p can aggravate myocardial I/R injury by regulating HMCN1 or cIAP1 [36]. There are also related studies reporting the critical role of these TFs (Nfkb1, Rela, Sp1, and Usf2) in the process of MIRI [37,38]. On the basis of the drug-related datasets, 21 therapeutic drugs (such as ritonavir, staurosporine, simvastatin, lutein, aspirin, warfarin, and clofibrate) that regulate OSRGs were predicted. Chronic ritonavir treatment increases cardiac output after ischemia–reperfusion injury in C57Bl/6 mice [39]. Pravastatin protects the myocardium from MIRI by regulating the miR-93/Nrf2/ARE signaling pathway [40]. The protective effect of aspirin on myocardial reperfusion has also been confirmed [41]. Further studies are needed to investigate the molecular mechanisms of these identified molecules or drugs on MIRI.

GSE160516 was used to evaluate the relationship between differentially expressed OSRGs and reperfusion time. The results showed that Apoe expression decreased within 24 h of reperfusion and then increased. On the contrary, Arg1 increased first and then decreased. The expressions of Hmox1, Hp, Mmp14, and Sirpa were significantly different at 6 h after reperfusion. The expressions of Hmox1, Hp, and Mmp14 were increased, and the expression of Sirpa decreased first and then increased. It can be seen that different OSRGs have different expression patterns at reperfusion time, and interventions targeting corresponding genes need to be carried out at different times. Further exploration of the dynamics of these genes and their relationship to the timing of intervention is needed.

OSRGs (Aif1, Apoe, Arg1, Col1a1, Gpx7, and Hmox1) were identified in the external dataset and the H9C2 cardiomyocyte H/R model. Enrichment analysis showed that these genes were mainly involved in the “response to oxidative stress” (Gpx1, Gpx7, Hmox1, Apoe), “negative regulation of smooth muscle cell proliferation” (Hmox1, Apoe, and Aif1), “response to hydrogen peroxide” (Col1a1, Gpx1, and Hmox1), and “lipoprotein particle binding” (Trem2 and Apoe) pathways. Aif1 promotes inflammation and OS in diabetic nephropathy through the miR-34a/ATG4B pathway [42]. Aif1 can also be activated during ischemia–reperfusion in rat liver to aggravate injury [43]. This suggests that Aif1 may be involved in MIRI in a similar mechanism. Apoe is one of the apolipoproteins playing an important role in lipoprotein metabolism and is a key factor to determine the level of lipid content in plasma. It can also be an important indicator for early diagnosis of coronary artery disease [44]. It has been shown that Apoe deficiency causes the formation of excess neutrophil extracellular traps and exacerbates myocardial damage in myocardial infarction mice through the OS process [45]. Arg1 is a key element in the urea cycle, converting L-arginine to urea and L-ornithine, which are further metabolized into metabolites proline and polyamide, respectively, driving key bioenergy pathways in collagen synthesis and cell proliferation [46]. Arg1 has been reported to play a protective role in retinal ischemia–reperfusion injury by inhibiting macrophage inflammation and OS [47]. Gpx7 is an antioxidant enzyme that protects the esophageal epithelium from hydrogen peroxide-induced OS, inhibits acidic bile acid-induced ROS generation, and prevents oxidative DNA damage and double-strand breaks [48]. The mechanism of Gpx7 in MIRI still lacks relevant research. Hmox1 is an antioxidant gene that relies on its ability to catabolize free heme, preventing it from making cells undergo apoptosis [49]. The antioxidant effect and protective role of Hmox1 in MIRI have been demonstrated [50,51]. Therefore, these identified genes may play an important role in MIRI by affecting OS, and further studies are needed to explore the exact mechanism.

This study was based on published datasets and cell experiments, which had some limitations. In vivo animal experiments and large sample multicenter clinical trials are needed to further validate the expression pattern and molecular mechanisms of these identified genes to explore better diagnostic and therapeutic approaches for MIRI.

## 5. Conclusions

In this study, MIRI-related transcriptome microarray datasets were analyzed using bioinformatics methods, the key pathways and hub genes of MIRI were preliminarily explored, and six OSRGs (Aif1, Apoe, Arg1, Col1a1, Gpx7, and Hmox1) significantly associated with MIRI were further selected. These identified genes may provide new directions for studying oxidative stress pathways in MIRI.

## Data Availability

The datasets presented in this study (GSE160516, GSE61592, and GSE4105) can be found in the GEO database (https://www.ncbi.nlm.nih.gov/, accessed on 2 August 2022).

## Supplementary Materials

The following supporting information can be downloaded at https://www.mdpi.com/article/10.3390/jcdd10020079/s1: Supplementary File S1. Oxidative stress-related genes in MSigDB; Supplementary File S2. The primer sequence information of qPCR experiment; Supplementary File S3. Differentially expressed genes in GSE160516 and GSE61592; Supplementary File S4. Results of three algorithms (EPC, Degree, and Betweenness) predicting hub genes.

## Abbreviations

AMI	Acute myocardial infarction
BP	Biological process
CC	Cellular component
DAMP	Damage-associated molecular pattern
DEG	Differentially expressed genes
GEO	Gene expression omnibus
H2O2	Hydrogen peroxide
HR	Hypoxia–reoxygenation
IR	Ischemia–reperfusion
KEGG	Kyoto Encyclopedia of Genes and Genomes
LAD	Left anterior descending
MF	Molecular function
MIRI	Myocardial ischemia–reperfusion injury
MSigDB	Molecular signatures database
OS	Oxidative stress
OSRG	Oxidative stress-related gene
PCI	Percutaneous coronary intervention
PPI	Protein–protein interaction
ROS	Reactive oxygen species
TF	Transcription factor
