# Supplementary figures and images for: Expression Pattern and Molecular Mechanism of Oxidative Stress-Related Genes in Myocardial Ischemia–Reperfusion Injury
Source: J Cardiovasc Dev Dis. 2023 Feb 13;10(2):79. doi: 10.3390/jcdd10020079 (PMC9961140; doi:10.3390/jcdd10020079)

Supplementary S4. Results of three algorithms EPC, Degree, and Betweenness predicting Hub Genes.

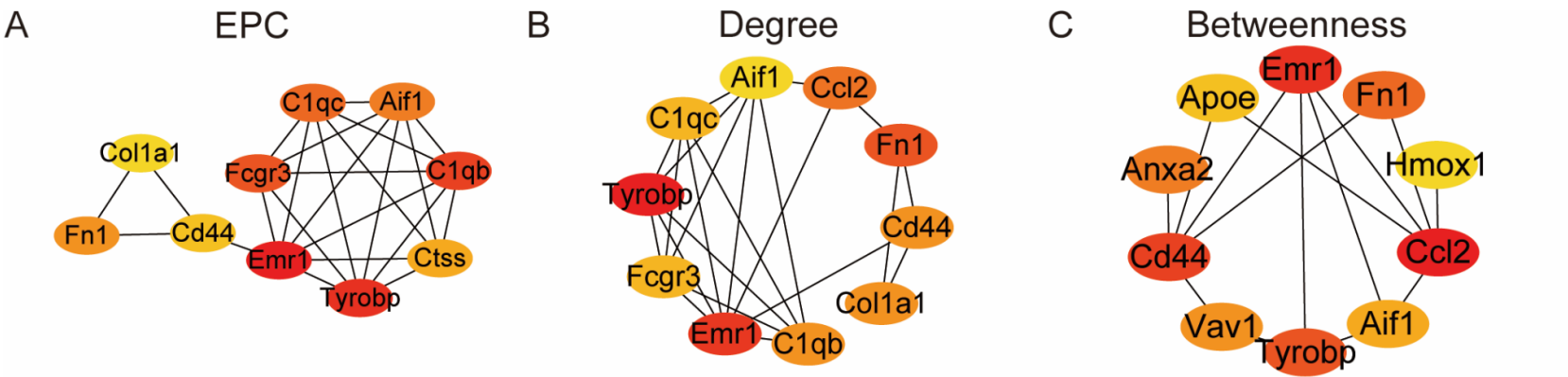

Supplement: Supplementary file 1 [file jcdd-10-00079-s001.zip › Supplementary File S4. Results of three algorithms (EPC, Degree, and Betweenness) predicting hub genes.pdf]
